# Acetylome in Human Fibroblasts From Parkinson's Disease Patients

**DOI:** 10.3389/fncel.2018.00097

**Published:** 2018-04-17

**Authors:** Sokhna M. S. Yakhine-Diop, Mario Rodríguez-Arribas, Guadalupe Martínez-Chacón, Elisabet Uribe-Carretero, Rubén Gómez-Sánchez, Ana Aiastui, Adolfo López de Munain, José M. Bravo-San Pedro, Mireia Niso-Santano, Rosa A. González-Polo, José M. Fuentes

**Affiliations:** ^1^Centro de Investigación Biomédica en Red en Enfermedades Neurodegenerativas, Madrid, Spain; ^2^Departamento de Bioquímica y Biología Molecular y Genética, Facultad de Enfermería y Terapia Ocupacional, Universidad de Extremadura, Cáceres, Spain; ^3^Department of Cell Biology, University of Groningen, University Medical Center Groningen, Groningen, Netherlands; ^4^Cell Culture Plataform, Donostia University Hospital, San Sebastián, Spain; ^5^Neuroscience Area of Biodonostia Health Research Institute, Donostia University Hospital, San Sebastián, Spain; ^6^Department of Neurology, Donostia University Hospital, San Sebastian, Spain; ^7^Ilundain Fundazioa, San Sebastian, Spain; ^8^Department of Neurosciences, University of the Basque Country UPV-EHU, San Sebastián, Spain; ^9^Equipe 11 labellisèe Ligue Contre le Cancer, Centre de Recherche des Cordeliers, Paris, France; ^10^INSERM U1138, Paris, France; ^11^Université Paris Descartes/Paris V, Sorbonne Paris Cité, Paris, France; ^12^Université Pierre et Marie Curie/Paris VI, Paris, France; ^13^Gustave Roussy Comprehensive Cancer Institute, Villejuif, France

**Keywords:** acetylation, LRRK2, peptides, Parkinson, proteins

## Abstract

Parkinson's disease (PD) is a multifactorial neurodegenerative disorder. The pathogenesis of this disease is associated with gene and environmental factors. Mutations in leucine-rich repeat kinase 2 (LRRK2) are the most frequent genetic cause of familial and sporadic PD. Moreover, posttranslational modifications, including protein acetylation, are involved in the molecular mechanism of PD. Acetylation of lysine proteins is a dynamic process that is modulated in PD. In this descriptive study, we characterized the acetylated proteins and peptides in primary fibroblasts from idiopathic PD (IPD) and genetic PD harboring G2019S or R1441G *LRRK2* mutations. Identified acetylated peptides are modulated between individuals' groups. Although acetylated nuclear proteins are the most represented in cells, they are hypoacetylated in IPD. Results display that the level of hyperacetylated and hypoacetylated peptides are, respectively, enhanced in genetic PD and in IPD cells.

## Introduction

Parkinson's disease (PD) is a disabling neurological disorder that is in progressive evolution. This neurodegeneration mainly affects the dopaminergic neurons of the *susbtancia nigra pars compacta* that are in part responsible for clinical motor symptoms. The widespread of this neurodegeneration from the midbrain to other neurotransmitter (serotoninergic and noradrenergic) systems elicits the appearance of non-motor symptoms (Politis and Loane, 2011; Deusser et al., 2015). Although well not understood, the etiopathogenesis of PD has thought related to environmental factors and gene mutations. Almost 90% of PD cases are sporadic (Ammal Kaidery et al., 2013), and may due to the susceptibility of genetic predisposition to environmental factors (Yakhine-Diop et al., 2014). Mutations in leucine-rich repeat kinase 2 (LRRK2) have been associated with autosomal dominant PD and involved in familial and idiopathic cases. Among six pathogenic LRRK2 mutations (R1441G/C/H, Y1699C, G2019S, and I2020T), the most frequent is the G2019S that affect almost 5% of familial PD and 2% of sporadic PD (Li et al., 2014). G2019S and R1441G are located, respectively, in the kinase and GTP domains of LRRK2 protein. Both mutations participate in the pathogenicity of PD through the impairment of LRRK2 enzymatic activity (Martin et al., 2014).

Evidence has reported that PARK genes, among other *synuclein* (*SNCA*) and *LRRK2*, regulate epigenetic mechanisms, thereby modulate gene expression (Coppedè, 2012). Indeed, gene expression is altered through posttranslational modifications of histones (methylation, phosphorylation, ubiquitination, and acetylation) and affects individual phenotypes (Ammal Kaidery et al., 2013). Parkinsonism-related toxins modulate histone acetylation by either decreasing histone deacetylase (HDAC) activity (Song et al., 2011) or increasing histone acetyltransferase (HAT) activity (Song et al., 2010). Studies, in PD post mortem brains, have reported that histone acetylation is upregulated in midbrain neurons; however, in some patients, this acetylation level varies differentially according to the brain regions and cell types (Park et al., 2016).

Acetylation is the transfer of an acetyl group from acetyl coenzyme A to the ε-amino group of lysine residues in proteins (histone and non-histone proteins) or to the α-amino group of the N-terminus of proteins. N-terminal acetylation is an irreversible reaction that is catalyzed by N-terminal acetyltransferases, whereas lysine acetylation is regulated by the balance of two enzymes HAT and HDAC (Drazic et al., 2016). The combination of these both acetylation reactions constitutes the acetylome. Acetylome in PD models is poorly characterized, only the variation of histone acetylation has been widely reported. Given that it may have an important role in PD pathogenesis, we identified some of those proteins that are acetylated in PD-associated (G2019S and R1441G) *LRRK2* mutations and idiopathic PD. Importantly, there are more acetylated peptides in genetic PD models and acetylated proteins are mainly nuclear and cytosolic.

## Materials and methods

### Cell culture

Fibroblasts from PD patients (with or without *LRRK2* mutations) and from control subjects were provided by Dr. Adolfo López de Munaín. Experiments were performed using four cell lines: Control (Co, patients who did not develop PD), IPD (IPD, PD patients without *LRRK2* mutations), GS (PD patients with G2019S *LRRK2* mutation), and RG (PD patients with R1441G *LRRK2* mutation). This study was carried out in accordance with the recommendations of Comité Ético de Investigación Clínica del Área Sanitaria de Gipuzkoa. All subjects gave written informed consent in accordance with the Declaration of Helsinki. Cells were grown in Dulbecco's modified Eagle's medium (DMEM, Sigma-Aldrich, D6546) supplemented with 10% of fetal bovine serum (FBS, Sigma-Aldrich, F7524), 1% L-glutamine (Sigma-Aldrich, G7513) and 2 mL streptomycin/penicillin (HyClone, Thermo Fisher Scientific, SV30010) at 37°C in 5% CO_2_/95% air. To confirm the G2019S or R1441G *LRRK2* mutations in cells, DNA was extracted (Macherey-Nagel Kit, 740952.50) and sequenced at STAB VIDA (Caparica, Portugal). In this study, we worked with pooled cell lines ranging from three to four cell lines (Table 1). Human fibroblasts (HFs) were seeded at a density of 3.5 × 10^4^ cells/mL and from lower passages.

**Table 1:** Presentation of the four groups of pooled cell lines.

**Groups**	**Names**	**Dates of Birth**	**Genotypes**
	Co1		LRRK2 WT
Co	Co2	1956–1977	LRRK2 WT
	Co3		LRRK2 WT
	Co4		LRRK2 WT
	IPD1		LRRK2 WT
IPD	IPD2	1928–1954	LRRK2 WT
	IPD3		LRRK2 WT
	GS1		G2019S Heterozygous
GS	GS2	1945–1949	G2019S Heterozygous
	GS3		G2019S Heterozygous
	RG1		R1441G Heterozygous
RG	RG2	1931–1942	R1441G Heterozygous
	RG3		R1441G Heterozygous

*The control group consists of four individuals, the IPD, GS and RG groups of three individuals each. We present in this table the range age of each group, and the genotype of each individual*.

### Digestion and desalting of peptides

Samples (Co, IPD, GS, and RG) were resuspended in 400 μL of 50 mM ammoniun bicarbonate and quantified by Bradford protein assay (BioRad). For each sample, 2 mg protein were diluted in 8 M Urea in-solution trypsin digestion. Proteins were reduced, alkylated, and digested with a 1:20 (w /w) ratio of recombinant trypsin sequencing grade (Roche) overnight at 37°C. Peptides from digested proteins were desalted and concentrated with a C18 reversed phase chromatography (ZipTip C18, Millipore) and the peptides were eluted in 50% acetonitrile (ACN)/0.1% trifluoroacetic acid (FA). Finally, the samples were freeze-dried in SpeedVac and dissolved in 200 μL of NETN (100 mM NaCl, 1 mM EDTA, 50 mM Tris pH 8, and 0.5% Nonidet P40) buffer for affinity enrichment of lysine-acetylated peptides.

### Enrichment of lysine-acetylated peptides

Samples in NETN buffer were incubated with anti-acetyl lysine agarose beads (catalog no. PTM-104, PTM Biolabs) at 4°C overnight with gentle shaking. After incubation, the beads were carefully washed three times with NETN buffer, twice with ETN buffer (1 mM EDTA, 50 mM Tris pH 8, and 100 mM NaCl), and once with water. The immunoprecipitated peptides were eluted with 1% FA and dried in a SpeedVac. The resulting peptides were cleaned with C18 Zip Tips (Millipore) according to the manufacturer's instructions and were dissolved in 12 μL of 2% ACN/0.1% FA then subjected to LC–MS/MS analysis by Triple TOF 5600 (SCIEX).

### Protein identification

MS/MS data sets were identified using Mascot licensed version 2.3.02 (Matrix Sciences) and ProteinPilot (revision 4895; AB SCIEX 5.0.1) using the Paragon algorithm (5.0.1.0, 4874). All data files were searched using the SwissProtHuman 2015_09_17 database with 42136 sequences. Search parameters in Mascot for acetylated peptides were as follows: trypsin digestion with five missed cleavages to account the inability of trypsin to cleave at acetylated lysine residues. Lysine acetylation, N-terminal acetylation and methionine oxidation were set as variable modifications, and carbamidomethyl cysteine as a fixed modification. Precursor ion and fragment ion mass tolerances were set to 30 ppm and 0.6 Da, respectively. Further, the decoy database search (Mascot integrated decoy approach) was used to false decoy recovery (FDR) calculation and the percolator algorithm applied to Mascot results. The acceptances criteria for proteins identification were a FDR < 1% and at least one peptide identified with a confidence interval (CI > 95%). In ProteinPilot, the following sample parameters were used: trypsin digestion, cysteine alkylation with iodoacetamide, and acetylation emphasis. A thorough identification (ID) search was done. Thus, a local FDR of 1% was chosen using the ProteinPilot FDR analysis tool (PSPEP) algorithm and a peptide CI value of 95%.

### Protein relative quantification

For human proteins relative quantification in (Co, IPD, GS, and RG) samples, the Raw profile data files (.raw) were imported into Progenesis LC-MS for proteomics (64-bit version v 4.1; Nonlinear Dynamics/Waters). Imported runs were chromatographic aligned to the reference run identified by the software. All runs were selected for peak picking with the automatic sensitivity method (default settings) and filtered to include only peaks with a charge state between 2 and 5. All detected features were normalized against the reference run by Progenesis LC-MS. Between-subject comparison was used as experimental design (Co, IPD, GS, and RG). Spectral data from selected features (*p*-value < 0.05) were transformed to Mascot generic format (MGF) files with Progenesis LC-MS and exported for peptide/protein identification to Mascot search engine, using the searched parameters above described. Mascot search results, that exceed the acceptance criteria for identification (FDR < 1%, peptides with individual ion scores >13, *p* < 0.05), were imported into Progenesis LC-MS as XML files and analyzed according to the following criteria: only were used quantitation from non-conflicting peptides. For each protein, the number or reported peptides was determined by counting unique peptide sequence. Only proteins reported by one or more peptides with a *p*-value < 0.05 were quantified.

Protein abundance was calculated from the sum of all unique normalized peptide ion abundance. Protein reported abundance is the geometric mean of the biological replicates. Proteins with a likelihood of quantification smaller than 0.05 (Anova *p*-value) were considered to be significantly regulated. Normalized peptide intensities were used to calculate fold-changes between samples. Relative abundance of human proteins (fold change) in three conditions compared with corresponding proteins in control samples were quantified by the ratio of summed peptide ion normalized abundance in each group to evaluate the enrichment of the protein. Differentially expressed proteins (*p* < 0.05) were considered with a fold change ≥1.3 and at least 1 identified peptides in at least one of replicates.

### Immunofluorescence

HFs were seeded on 96-well plate at a density of 3500 cells/well. Cells were successively fixed with 4% PFA and permeabilized with 0.1% Triton (Sigma-Aldrich, T9284) for 20 and 5 min, respectively, at room temperature (RT). Once permeabilized, plated cells were incubated with bovine serum albumin (BSA)/PBS solution (1 mg/mL) for 1 h at RT and then with the primary antibody acetyl-H4 (G-2) (Ser1K5K8K12) (1:50, Santa Cruz Biotechnology, sc-393472), while shaking overnight at 4°C. The following day, cells were reincubated with Alexa Fluor® 568 (1:100 Thermo Scientific, A11004)-conjugated secondary antibodies for 1 h at RT. Nuclei were stained with Hoechst 33342 (2 μM, Sigma Aldrich, B2261). Images were visualized using an Olympus IX51 inverted microscope.

### Statistical analyses

Statistical analyses were assessed by Student's *t*-test, Chi-Squared test or Anova test. The results were considered significant at *p*<0.05.

## Results

### Detection of acetylated proteins

**Figure 1:**
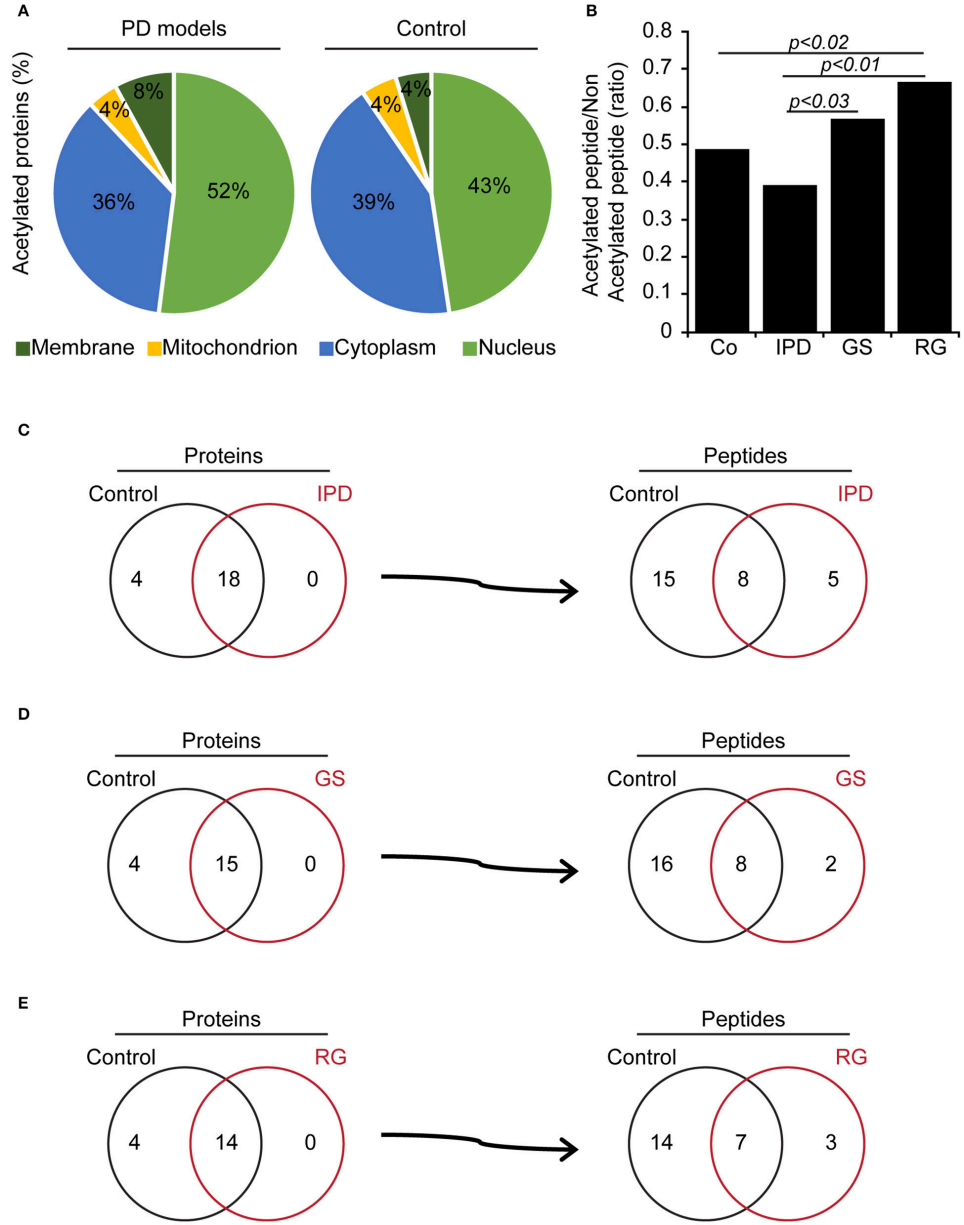
Acetylated proteins in human fibroblasts. **(A)** Subcellular location of acetylated proteins (%) in human fibroblasts. **(B)** Ratio of identified acetylated peptides/non-acetylated peptides in human fibroblasts, *p* < 0.03, *p* < 0.02 and *p* < 0.01 compared to control (χ^2^-test). **(C–E)** Comparison of acetylated proteins and identified acetylated peptides per proteins between Co line and PD models. **(C)** Co and IPD lines, **(D)** Co and GS lines **(E)** Co and RG lines represent the acetylome of each group and what they share in common.

We determined the N-terminal acetylation and acetylated lysine (Ac-K) proteins in fibroblasts from PD patients and control subjects. In Tables 2, 3, we have listed some of those acetylated proteins. Some of them (fructose bisphosphate aldolase C (ALDOC), Glyceraldehyde-3-phosphate dehydrogenase (GAPDH), Alpha-Enolase, pyruvate kinase) are enzymes that participate at different steps of glycolysis pathway (TeSlaa and Teitell, 2014). The rest of acetylated proteins are implicated in cell proliferation, acetylation, apoptosis and nucleosome wrap, as well in the link between glycolysis and citric acid cycle. Most of those proteins were located in the nucleus and the cytoplasm and represent, respectively, 52 and 36% of acetylated proteins in PD patients (Figure 1A). The less represented were found in plasma membrane (8%) and mitochondrion (4%).This distribution did not change in the control group but the proportion slightly differs. The number of Ac-K sites on proteins varied from one to ten (Tables 2, 3) and can be found on distinct peptides. Furthermore, in PD models some acetylated proteins had more or less Ac-K sites than Control line (Table 2). Interestingly, we found that the ratio of acetylated peptides/non-acetylated peptides was enhanced in GS and RG cells while it was decreased in IPD cells (Figure 1B). We inferred that there were more acetylated peptides levels in familial PD than in sporadic PD. These variations were significant between genetic and idiopathic PD.

**Table 2:** Acetylated proteins in fibroblasts from PD patients with or without LRRK2 mutation.

**Protein ID**	**Protein Name**	**Subcellular Location**	**Ac-K Sites**
P07355	Annexin A2	Extracellular space, Extracellular matrix	10
P04083	Annexin A1	Nucleus, Cytoplasm, Cell membrane	1
P08670	Vimentin	Cytoplasm	2
Q9BQE3	α-Tubulin 1C	Cytoplasm, Cytoskeleton	1
P68363	α-Tubulin 1B	Cytoplasm, Cytoskeleton	1
P14618	Pyruvate kinase	Cytoplasm, Nucleus	2
Q6PEY2	α-Tubulin 3E	Cytoplasm, Cytoskeleton.	1
P08758	Annexin A5	–	4
P21333	Filamin-A	Cytoplasm, Cell cortex, Cytoskeleton.	1
P62805	Histone H4	Nucleus, Chromosome	4
Q9GZZ1	NAA50	Cytoplasm	2
Q09472	HAT p300	Cytoplasm, Nucleus.	5
Q92793	CBP	Cytoplasm, Nucleus.	2
Q99880	H2B1L	Nucleus, Chromosome	4
P04406	GAPDH	Cytoplasm, Cytoskeleton	4
P68371	β-Tubulin 4B	Cytoplasm, Cytoskeleton, Nucleus	1
P07437	β-Tubulin	Cytoplasm, Cytoskeleton.	1
P06733	Alpha-enolase	Cytoplasm, Cell membrane	4
O43809	CPSF5	Nucleus	1
P62328	Thymosin β-4	Cytoplasm, Cytoskeleton.	3
P22392	NME2	Cytoplasm, Nucleus	2
Q15942	Zyxin	Cytoplasm, Cytoskeleton, Nucleus, Cell junction	2
P21796	VDAC1	Outer mitochondrial membrane	1
P20962	Parathymosin	Nucleus	4
P08559	PDHA1	Mitochondrial matrix	4
P06454	Prothymosin α	Nucleus	5
P68871	Hemoglobin β	–	1
P02042	Hemoglobin Δ	–	1
P09972	ALDOC	–	1
P04792	Heat shock protein beta-1	Cytoplasm, Nucleus	1
P06703	Protein S100-A6	Nucleus envelope, Cell membrane	1
P00338	LDHA A	Cytoplasm	4

*We specified the subcellular location of proteins and the number of acetylation sites. Lines filled in blue represent the most relevant variations identified in PD models compared to control*.

**Table 3:** Acetylated proteins in human fibroblasts from control subjects.

**Protein ID**	**Protein Name**	**Subecellular Location**	**Ac-K Sites**
P07355	Annexin A2	Extracellular space, Extracellular matrix	10
P04083	Annexin A1	Nucleus, Cytoplasm, Cell membrane	2
P08670	Vimentin	Cytoplasm	4
Q9BQE3	Tubulin α-1C	Cytoplasm, Cytoskeleton	2
P08758	Annexin A5	–	3
P21333	Filamin-A	Cytoplasm, Cell cortex, Cytoskeleton.	1
P62805	Histone H4	Nucleus, Chromosome	3
Q9GZZ1	NAA50	Cytoplasm	2
Q09472	HAT p300	Cytoplasm, Nucleus.	1
Q92793	CBP	Cytoplasm, Nucleus.	2
Q99880	H2B1L	Nucleus, Chromosome	4–7
P04406	GAPDH	Cytoplasm, Cytoskeleton	2
P68371	Tubulin β-4B	Cytoplasm, Cytoskeleton, Nucleus	1
P07437	Tubulin β	Cytoplasm, Cytoskeleton.	1
P06733	Alpha-enolase	Cytoplasm, Cell membrane	4
O43809	CPSF5	Nucleus	1
P62328	Thymosin β-4	Cytoplasm, Cytoskeleton.	2
P22392	NME2	Cytoplasm, Nucleus	2
Q15942	Zyxin	Cytoplasm, Cytoskeleton, Nucleus, Cell junction	2
P21796	VDAC1	Outer mitochondrial membrane	1
P20962	Parathymosin	Nucleus	2
P06454	Prothymosin α	Nucleus	4
P68871	Hemoglobin β	–	1
P02042	Hemoglobin Δ	–	1
P09972	ALDOC	–	1
P06703	Protein S100-A6	Nucleus envelope, Cell membrane	1
P00338	LDHA A	Cytoplasm	4
P04075	ALDOA	–	1
P08133	ANXA6	Cytoplasm	4
P06748	NPM	Cytoplasm, Nucleus	1
Q8NCA9	ZN784	Nucleus	1
P33778	H2B1B	Nucleus	3
P16401	Histone1.5	Nucleus	2

*The subcellular location of proteins and the number of Ac-k sites*.

### Identification of acetylated peptides and proteins in PD fibroblasts

**Figure 2:**
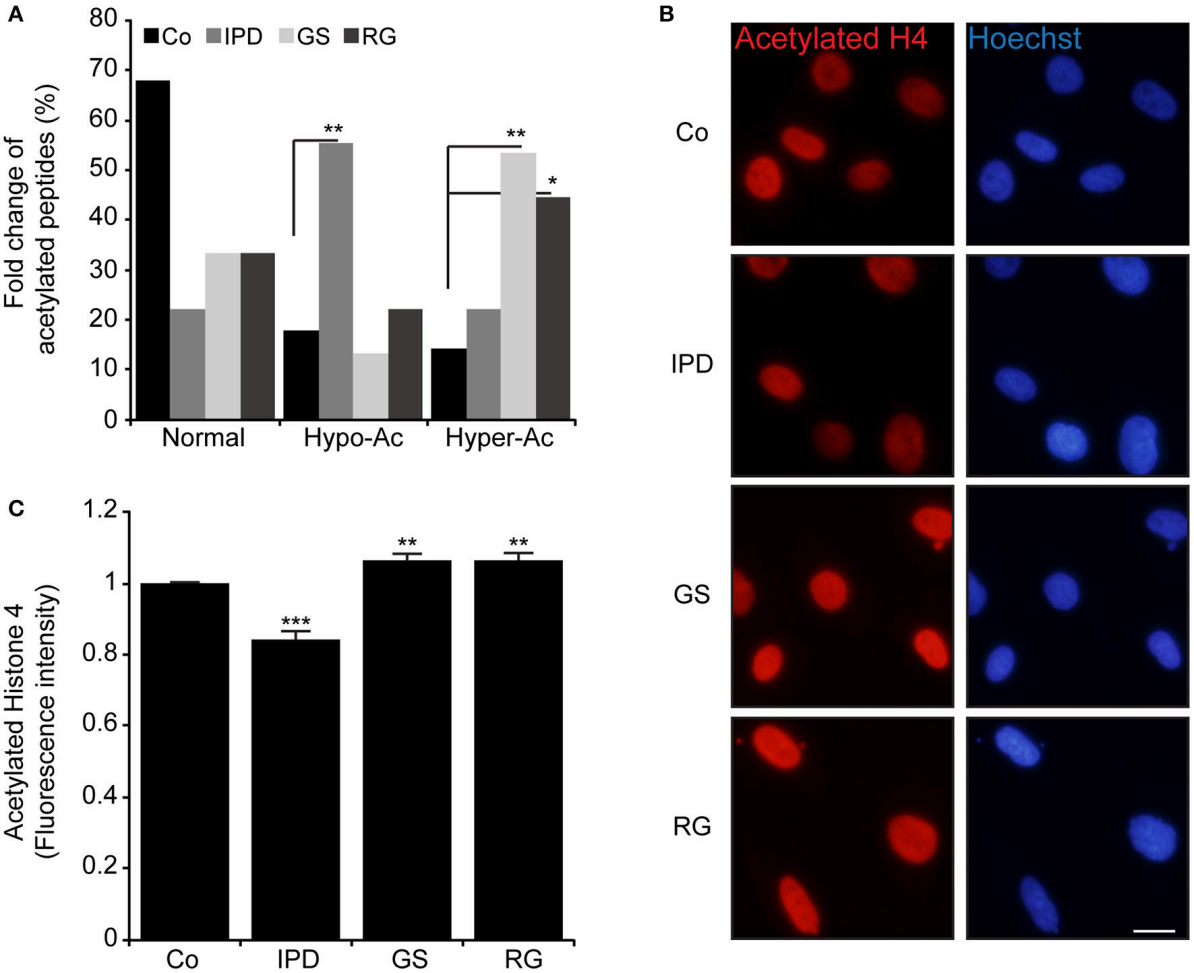
Modulation of acetylated peptides in human fibroblast **(A)** Represents in % the fold change of hypoacetylated and hyperacetylated peptides in HFs compared to Co, ^*^p < 0.05 and ^**^p < 0.01 (χ^2^-test). **(B,C)**. Acetylated histone 4 (Ac-H4K5K8K12) (red) was detected by immunofluorescence and the nuclei were stained with Hoechst 33342 (blue), Original magnification: 20X, scale bar corresponds to 10 μm. **(C)** Represents the quantification of fluorescence intensity of labeled Ac-H4K5K8K12 by imageJ (*n* = 200 cells). Data represent the mean ± SEM of at least three independent experiments, ^**^p < 0.01 and ^***^p < 0.001 respect to Co (Student's *t*-test).

To elucidate these differences, PD patients' data were compared to Co data. We observed that there were four acetylated proteins only belonged to Co (Figures 1C–E), however two of them (Nucleophosmin and Zinc finger protein 784) were constant. The pairs of thymosin β4 and H2B1B or H2B1B and H2B1M or Annexin A6 and Histone 1.5 were additionnally found in Co line when compared to IPD, GS, and RG lines, respectively. Moreover, Co and PD lines have acetylated proteins in common. From the detected acetylated proteins based on the comparison effectuated respect to Co line, distinct acetylated peptides were identified and were characteristic of each group. Thus, 2 out of 5 acetylated peptides [**S**TVHEILCK (Annexin A2 protein) and **K**GS**KK**AVT**K**AQKK (H2B1L protein)] are specific to IPD line, 2 to GS line [FLEQQN**K**ILLAELEQLK (Vimentin protein) and **K**GS**KK**AVTK (H2B1C protein)] and 1 out of 3 to RG line [GVTQFGN**K**YIQQTK (CPSF5 protein)]. Of note, proteins can be acetylated in one or more peptides, and following the group of healthy subjects or PD patients, the acetylation of one protein might change from one peptide to another and be monoactylated, polyacetylated or inexistent. Given that the fold change of acetylated peptides depend on the relative abundance of proteins, the level of acetylated peptides was classified in three categories (Normal, hypoacetylated, and hyperacetylated). Therefore, it exists more hypoacetylated peptides in IPD line than in Co line. Also, the percentage of hyperacetylated peptides was significantly increased in cells harboring LRRK2 mutations (Figure 2A). By immunofluorescence staining, we observed that the intensity of acetylated Histone 4 was significantly reduced in IPD line (Figures 2B,C).

## Discussion

Dysregulation of acetylation machinery can lead to neurodegenerative diseases. In fact, protein aggregation is a hallmark of neurodegeneration and it is regulated by protein acetylation including lysine acetylation (Lee and Finkel, 2009) and N-terminal acetylation. It has been reported that the N-terminal acetylation of SNCA prevents its aggregation by stabilizing protein formation (Bartels et al., 2014), besides that, N-terminal acetylation can also act as a recognition tag to mediate protein degradation (Zattas et al., 2013), thus N-acetylation deficiency could be associated with PD pathogenesis. Despite this protective effect, N-terminal acetylation can disturb further proteasome degradation interplaying the N-terminal ubiquitylation of α-amino group of proteins substrates (Tatham et al., 2013).

Even though 80% of human proteins are acetylated at their N-terminal, they are poorly considered (Aksnes et al., 2016). In this study, the proteins N-terminally acetylated in PD models are annexin A2, putative annexin A2-like protein, cytosolic (Thymosin β4, α-enolase), nuclear (parathymosin, protein S100-A6, prothymosin α), and participate in various cellular processes. These proteins are more represented in IPD line. In common to IPD and GS lines, prothymosin α (Qi et al., 2010) and protein S100-A6 (Bartkowska et al., 2017) are, respectively, anti-apoptotic and stress modulator. α-Enolase is found in RG line whereas annexin A2 is in all PD models. Annexins (A1, A2, and A5) are calcium sensors that translocate to plasma or nuclear membrane (Skrahina et al., 2008) and affect apoptosis pathways (Debret et al., 2003; Jiang et al., 2015). It remains to investigate whether the N-acetylated form of these proteins have a crucial role in the progression of PD. Acetylated thymosin β4 is common to both genetic PD lines, this protein has a critical role in actin polymerization (Mannherz et al., 2010). Moreover, *LRRK2* mutations influence cytoskeleton organization. In fact, GTP LRRK2 domain interacts with β-tubulin and increases the lysine acetylation of α-tubulin. However, this interaction is altered with G2019S and R1441G *LRRK2* mutations and affects the stability of microtubules (Law et al., 2014). Indeed, neither microtubule structure nor conformation has affected by α-tubulin acetylation but influences the tubulin-binding proteins therefore tubulin functions (Howes et al., 2014). Damaged mitochondria-induced reactive oxygen species (ROS) are responsible for α-tubulin hyperacetylation (Bonet-Ponce et al., 2016). Such modification is required for an adaptive cell response through autophagy induction, consequently, this hyperacetylation is negatively regulated by p300 upon stress (Mackeh et al., 2014). In G2019S *LRRK2* mutation, the reduction of mitochondrial membrane potential is accompanied by an increase of ROS production and an enhancement of the basal autophagy level (Yakhine-Diop et al., 2014). An impairment of autophagy induction was also observed in IPD and R1441G LRRK2 lines (Data not shown). The HATs p300 and CBP are acetylated in RG line, respectively, at five and 2 lysine positions, which means p300 basal activity is increased (Drazic et al., 2016). Even though, HAT and HDAC are involved in the modulation of protein acetylation, acetyl-CoA availability is critical. In mammals, acetyl CoA is in part generated from pyruvate by pyruvate dehydrogenase (PDHA1). Pyruvate is the final product of glycolysis pathway (Drazic et al., 2016). Some enzymes of this pathway are lysine acetylated in IPD, GS, and RG lines. This posttranslational modification can increase or decrease the enzymatic activity of certain proteins. In the case of acetylated PDHA1 in RG line, its activity is decreased (Drazic et al., 2016), therefore the level of acetyl-CoA formation from glycolysis may be reduced. The different pathways (glycolysis and fatty acid β-oxidation) generating acetyl CoA interplay in mitochondria. Generally, mitochondria are defective in PD, this dysfunction disturbs the acetylation machinery by reducing HDAC Class III (sirtuins) activity (Schwab et al., 2017). Additionally, an imbalance between HDAC and HAT activities lead to hyperacetylation (Park et al., 2016) or hypoacetylation of proteins. Taken together, proteins are acetylated in IPD and LRRK2 mutations-associated PD. However, it occurs more hyperacetylated proteins in cells harboring LRRK2 mutations than in IPD lines. This variation seems to be associated with the disease. Moreover, in healthy subjects harboring the R1441G *LRRK2* mutation, the intensity of acetylated proteins was enhanced (Data not shown). The molecular mechanism of protein acetylation in PD remains unclear. It will be interesting to elucidate how proteins can be hypoacetylated in IPD rather than in Genetic PD.

## Author contributions

JF conceived the project. SY-D, JB-S, RG-S, MN-S, RG-P, and JF designed the experiments. SY-D, MR-A, GM-C, and EU-C performed experiments. MR-A performed the statistical analyses. Authors assisted in data analysis and interpretation. AA and AL performed the human fibroblast biopsies and culture. SY-D and JF wrote the manuscript. All authors revised and approved the content of the manuscript for publication.

### Conflict of interest statement

The authors declare that the research was conducted in the absence of any commercial or financial relationships that could be construed as a potential conflict of interest.

## Abbreviations

CI: Confidence interval
FA: Trifluoroacetic acid
FDR: False decoy recovery
GAPDH: Glyceraldehyde-3-phosphate dehydrogenase
H2B: Histone 2B
HAT: Histone acetyltransferase
HDAC: Histone deacetylase
ID: Identification
IPD: Idiopathic Parkinson's disease
K: Lysine
LC-MS: Liquid chromatography-mass spectrometry
LRRK2: Leucine-rich repeat kinase 2
PD: Parkinson's disease
PDHA1: Pyruvate dehydrogenase
PSPEP: Proteomics System Performance Evaluation Pipeline Software
ROS: Reactive oxygen species.
